# Gene annotation errors are common in the mammalian mitochondrial genomes database

**DOI:** 10.1186/s12864-019-5447-1

**Published:** 2019-01-22

**Authors:** Carlos F. Prada, Jeffrey L. Boore

**Affiliations:** 1 Departamento de Biología, Facultad de ciencias, Universidad del Tolima, Barrio Santa Helena Parte Alta, Ibagué, Colombia; 2 Providence St. Joseph Health and Institute for Systems Biology, 401 Terry Avenue N, Seattle, WA 98109 USA

**Keywords:** Gene rearrangement, Mammalian mitochondrial genome, Gene annotation, Comparative genomic analysis, Annotation errors

## Abstract

**Background:**

Although animal mitochondrial DNA sequences are known to evolve rapidly, their gene arrangements often remain unchanged over long periods of evolutionary time. Therefore, comparisons of mitochondrial genomes may result in significant insights into the evolution both of organisms and of genomes. Mammalian mitochondrial genomes recently published in the GenBank database of NCBI show numerous rearrangements in various regions of the genome, from which it may be inferred that the mammalian mitochondrial genome is more dynamic than expected. However, it is alternatively possible that these are errors of annotation and, if so, are misleading our interpretations. In order to verify these possible errors of annotation, we performed a comparative genomic analysis of mammalian mitochondrial genomes available in the NCBI database.

**Results:**

Using a combination of bioinformatics methods to carefully examine the mitochondrial gene arrangements in 304 mammalian species, we determined that there are only two sets of gene arrangements, one that is shared by all of the marsupials and another that is shared by all of the monotremes and eutherians, with these two arrangements differing only by the positions of tRNA genes in the region commonly designated as “WANCY” for the genes it comprises. All of the 68 other cases of reported gene rearrangements are errors. We note that there are also numerous errors of impossibly short, incorrect gene annotations, cases where genomes that are reported as complete are actually missing portions of the sequence, and genes that are clearly present but were not annotated in these records.

**Conclusions:**

We judge that the application of simple bioinformatic tools in the verification of gene annotation, particularly for organelle genomes, would be a very useful enhancement for the curation of genome sequences submitted to GenBank.

**Electronic supplementary material:**

The online version of this article (10.1186/s12864-019-5447-1) contains supplementary material, which is available to authorized users.

## Background

Mitochondria are semi-autonomous cytoplasmic organelles with their own DNA and ribosomes, found in all eukaryotic organisms except for a few groups of protozoans. Mitochondria play a variety of important roles, including the generation of ATP through oxidative phosphorylation and initiation and execution of apoptosis [1]. Mitochondria have their own genome (mtDNA), having evolved from that of their bacterial progenitors, with genes that participate in the energy currency of the cell and other processes. Therefore, variation in these genes can directly influence metabolic performance and its variation among different species [2].

The mitochondrial genome of animals, with only a few exceptions, is a small, circular genome, ranging from 15 to 20 Kb in size and containing genes for 13 proteins (*atp6, atp8, cob, cox1–3, nad1–6, nad4L*), 2 ribosomal RNA (rRNAs; *rrnS, rrnL*), and 22 transfers RNA (tRNAs; trnX, where X is the one letter code for the corresponding amino acid) [3]. For nearly all tetrapods, it also contains two main noncoding regions: (1) the control region (“CR”), containing a displacement loop (“D-loop”) structure and having several functional roles, including an origin of heavy-strand replication and promoters for transcription of each strand, and (2) the light-strand replication origin (O_L_) [4]. (The designation of the strands as heavy and light for tetrapod mtDNAs is based on differential sedimentation during centrifugation of the isolated strands because of differing base composition).

For many lineages, animal mitochondrial genomes have a relatively low rate of gene rearrangements compared to the nuclear genome [5], although there are some invertebrate lineages with radically rearranged mitochondrial gene orders (See some early discovered examples in [3]). Due to the recent increase in the number of sequenced mitochondrial genomes of hundreds of species, it has become clear that even some vertebrate lineages deviate with a modest number of gene rearrangements. For example, within tetrapods, gene rearrangements have been found for some species of lizards, amphibians, fish, crocodilians, snakes, tuatara, and lamprey [6–8]. Most of these rearrangements involve tRNA genes, *nad5*, and/or the D-loop region [6, 9–11].

Rearrangements of mitochondrial genes can have profound functional implications on gene expression and genome replication [12]. Further, the comparison of gene arrangements has been used to resolve a number of phylogenetic relationships that had been recalcitrant to all other methods [13], and have provided insight into the patterns and relative probabilities of various structural changes [14, 15]. Therefore, it is important that these gene arrangements be reported accurately for their interpretation in these contexts.

Mammalian mtDNAs have been particularly well investigated and initially were thought to be remarkably stable for genome rearrangements [3, 6]. However, dozens of rearrangements have now been reported in mammalian mitochondrial genomes, mainly in tRNAs. On casual inspection, many more such rearrangements appear in the GenBank records, which could be in error, especially since previous work has identified this to be troublingly common (see, for example, [16]) than have been described in the scientific literature (which is becoming ever more sparse in such descriptions as the pace of genome sequencing has greatly accelerated). Due to the possible physiological and adaptive impact of these rearrangements and addressing the utility of these in phylogenetic inference, we systematically investigated the gene annotation of the 304 complete mammalian mitochondrial genomes that are available in the GenBank database; with the aim of verifying correct gene annotations and identifying any errors it may contain. In so doing, we created a simple methodology that could be applied to correct current annotations and to identify any errors in new submissions.

## Methods

### Genomic sequences and multiple alignments

We retrieved the sequences and gene annotations of the 304 complete mammalian mitochondrial genomes, representing 29 taxonomic orders, that are available at the organelle genome resources database from NCBI (http://www.ncbi.nlm.nih.gov/genomes/OrganelleResource.cgi?opt=organelle&taxid=33208) as of September 15, 2017.

A total of 10 species of extinct mammals were included. Mitogenomes representing strains within the same species were not included (as in the case of the mouse, *Mus muscullus*, for which there are now mitogenome sequences for at least 20 individuals). Additional file 1: Tables S1 and Additional file 2: Table S2 list these species, sorted taxonomically, and provide the GenBank Reference IDs, reported gene arrangements (color-coded to results of these analyses; see below), and lengths of each mitochondrial gene and genome as annotated in these GenBank records.

The NCBI-BLAST2 sequence comparison program (https://blast.ncbi.nlm.nih.gov/Blast.cgi) was used to confirm rearrangements in the mitochondrial genome by comparing nucleotide sequences of orthologous regions from evolutionarily close species. We compared each reported translocation and inversion to the orthologous regions of closely related species in each orientation with 80% sequence identity as the threshold for determining the correct orientation, because lineages with frequent rearrangements also show elevated levels of nucleotide sequence variation, which leave a distinctive sequence footprints left by putative gene order rearrangements mainly observed in inversions [17]. We used Geneious software v 6.1.8 [18] to visualize these gene annotations and MUSCLE [19] for multiple sequence alignments. The tRNAscan-SE 2.0 program [20] was used to detect tRNA-encoding genes and confirm their orientation in the mitochondrial genomes, some of which were manually folded to verify conformity to expected secondary structures of their tRNA products.

## Results

According to the annotation of the 304 mammalian mitochondrial genomes found in the NCBI database, there are a total of 187 cases of genes annotated to be in arrangements differing from human mtDNA (a common gene arrangement shared also with monotremes and inferred by parsimony to be in the primitive state for mammals and other non-mammal groups, e.g. fish, or birds [3, 6]). These involve only tRNA genes, most within the “WANCY” region (that region containing the tRNA genes *trnW, trnA, trnN, trnC, trnY* that is between *nad2* and *cox1*), with rearrangements of *trnI-trnQ, trnE*, and *trnT-trnP* also being common. Of these 187 rearrangements, 128 (68%) are reported among marsupials, represented by the seven orders Microbiotheria, Paucituberculata, Notoryctemorphia, Diprotodontia, Dasyuromorphia, Peramelemorphia, and Diprotodontia (Table 1). The analysis of rearrangements in each of the mammalian mitochondrial genomes analyzed is summarized in Additional file 1: Table S1.

**Table 1:** Analysis of rearrangements in the mammalian mitochondrial genome reported in the NCBI database

	Taxonomic group	No. species	Genes differing in arrangement	Number confirmed	Number refuted	Endpoint errors noted	Present but not annotated	Genes or CR not present
	Monotremes	3	0	0	0	1	0	0
Marsupial	Didelphimorphia	4	16	16	0	0	1	4
	Paucituberculata	2	8	8	0	0	0	0
	Microbiotheria	1	4	4	0	0	0	3
	Diprotodontia	12	48	47	1	0	0	1
	Dasyuromorphia	6	32	24	8	0	0	0
	Notoryctemorphia	1	4	4	0	0	0	1
	Peramelemorphia	4	16	16	0	0	0	8
Eutheria	Afrosoricida	3	0	0	0	0	0	0
	Macroscelidea	2	0	0	0	0	0	0
	Tubulidentata	1	0	0	0	0	0	0
	Proboscidea	5	2	0	2	0	0	0
	Hyracoidea	2	1	0	1	0	0	0
	Sirenia	2	0	0	0	0	0	0
	Cingulata	1	0	0	0	0	0	0
	Pilosa	3	0	0	0	0	0	0
	Dermoptera	1	0	0	0	0	0	0
	Scandentia	1	0	0	0	0	0	0
	Primates	44	12	0	12	4	0	0
	Lagomorpha	6	0	0	0	0	0	0
	Rodentia	26	3	0	3	0	2	0
	Soricomorpha	5	2	0	2	0	0	0
	Erinaceomorpha	4	0	0	0	0	0	4
	Carnivora	65	9	0	9	3	0	1
	Pholidota	2	1	0	1	0	0	0
	Perissodactyla	9	0	0	0	0	0	0
	Artiodactyla	47	17	0	17	0	0	0
	Cetacea	32	12	0	12	1	0	0
	Chiroptera	10	0	0	0	0	0	0
	Total	304	187	119	68	9	3	22

Number of genes in mammalian mitochondrial genomes differing in position from that of humans (inferred to be the ancestral arrangement for all mammals) as reported in GenBank that were confirmed or refuted in this study, along with a summary of additional errors noted. See Additional file 1: Table S1 and Additional file 2: Table S2 for details. Each of the gene arrangement errors found brings into conformity a single shared gene arrangement for all of the marsupials and another shared gene arrangement that is shared among all monotremes and eutherians. On some mitogenomes, more than one annotation error was detected

**Fig. 1:**
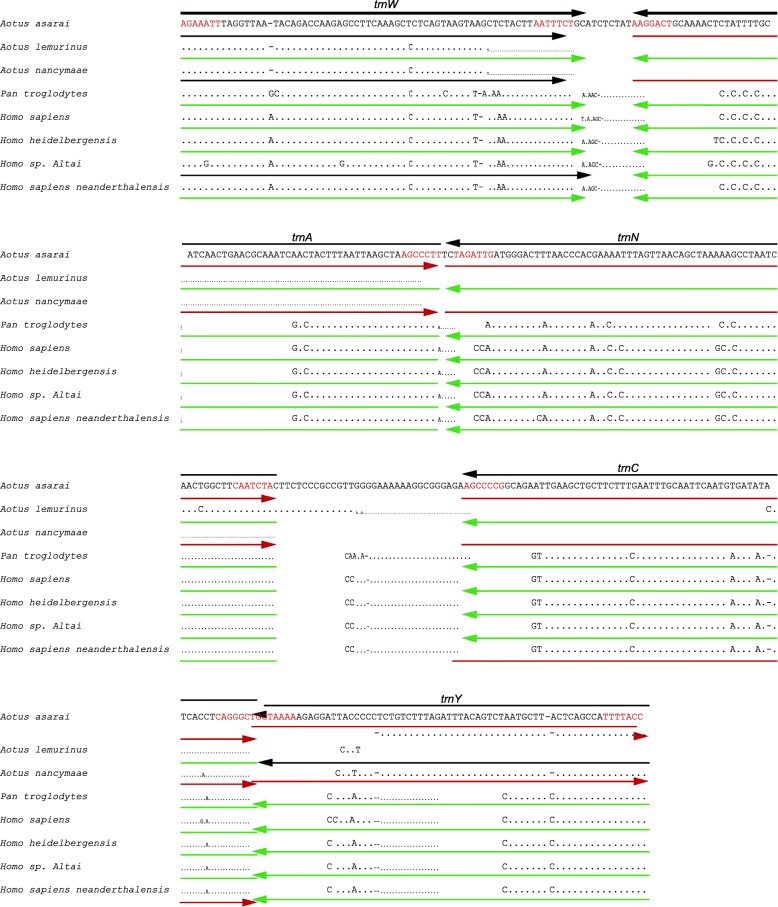
Multiple sequence alignment of the “WANCY region” in eight different primates. The analysis showing very high levels of sequence similarity throughout. The proper annotation of each tRNA gene as determined by manual folding of potential secondary structures is shown in black above the alignment. Nucleotides shown in red are those predicted to be paired in the amino-acyl acceptor arm of the mature tRNA. The annotations of these genes as they appear in the GenBank records is shown below each sequence with green representing conformity to the annotation inferred to be correct, brown representing errors in designating the endpoints of the genes, and red representing errors in gene orientation

The alignments made with the MUSCLE program show a high pairwise identity among homologous genes. For example, when analyzing the “WANCY” region of eight different primate species (some of which are annotated with inversions in these tRNA genes), from a total of 44 primates analyzed, the region that presents the highest concentration of reported inversions, the pairwise identity is greater than 93.1% (Fig. 1).

The gene order of most primates (including *Aotus lemurinus*) is confirmed as “W-A-N-C-Y”, but in *Aotus azarai* and *Aotus nancymaee* this is annotated as “WANCY”. (Here and hereafter, arrangements of tRNA genes will be indicated by the single-letter code for the corresponding amino acid and with a minus sign to indicate opposite strand orientation.) MUSCLE alignment shows a nucleotide pairwise identity of 98.3% among the three *Aotus* species and, as confirmed by a reannotation using the tRNAscan-SE program, the reported arrangement of “WANCY” is clearly incorrect, presumably because those submitting sequences of *A. azarai* and *A. nancymaee* failed to properly designate these as being on the opposite strand. Other possible explanation is that these mitogenomes were obtained with an amplification strategy by PCR, sequencing and assembly [21], which could explain the possible gene annotation errors. A similar result is observed in *Homo sapiens neanderthalensis*, which is annotated with the “W-A-NC-Y” order (i.e., *trnC* is reversed in orientation), but is actually identical to the “W-A-N-C-Y” annotated in contemporary *Homo sapiens* as well as *Homo sapiens altai* (Fig. 1).

In this same region, the order of tRNAs is reported to be “-A-CW-N-Y” for 29 of the 30 marsupial mtDNAs in GenBank. In contrast, for *Dactylopsila trivirgata,* belonging to order Diprotodontia, this region is annotated as “A-CW-N-Y” (i.e., with *trnA* reading left-to-right as shown). The MUSCLE sequence alignment shows an average nucleotide pairwise identity of 98.3% for *trnA* in the 12 species of this order and, along with the results from NCBI-BLAST2 sequence and tRNAscan-SE, confirm that *D. trivirgata* is annotated in error and confirms that all 30 of these marsupial mtDNAs share the gene order “-A-CW-N-Y”, which is a shared rearrangement from the ancestral mammalian state (Additional file 3: Figure S1).

In nearly all mammalian species, the order I-Q (between *nad1* and *trnM* genes) is reported. However, in four species of the marsupial order Dasyuromorphia, two inversions are reported that generate the -IQ ordering. We verify using both tRNAscan-SE and sequence alignments that this order is erroneous, confirming the correct order as I-Q. In a similar way, annotation errors in the I-Q region were found in *Loxodonta africana* (Proboscidea), *Nyctereutes procyonoides* (Carnivora), *Manis tetradactyla* (Pholidota), *Bubalus bubalis, Capra hircus, Naemorhedus caudatus*, and *Vicugna pacos* (Artiodactyla), *Crocidura russula* (Soricomorpha), and *Feresa attenuate* (Cetacea). Inversions of tRNA-encoding genes are also erroneously reported for many cases within the “WANCY” region and for *trnE* (12 cases), *trnP* (9 cases), *trnS* (2 cases) and *trnT* (2 cases). See Tables S1 and S2.

In two rodent mtDNAs, those of *Castor canadensis* and *C. fiber*, there is no annotation of *nad6*; this gene is actually in the expected position with high sequence similarity to homologous genes of closely related species.

**Fig. 2:**
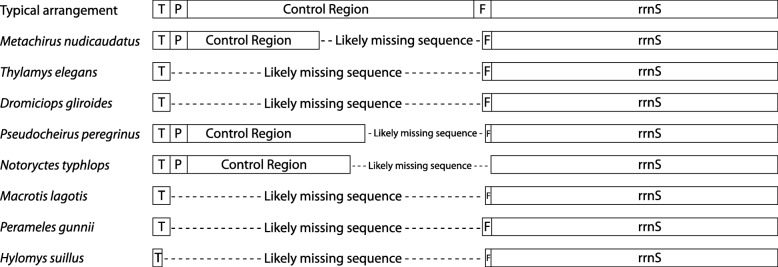
Arrangement of annotated sequences of the region *trnT-trnP*-CR-*trnF-rrnS* from the GenBank files. Analysis of eight mtDNAs inferred in this study to be incompletely sequenced (in contradiction to the statements in these files in GenBank that they are complete). Each region is drawn approximately to scale, illustrating that many genes are not full length and that intervening sequence appears to be missing

It has long been noted that there are often difficulties in determining the sequence of the CR (D-loop region), with speculation that this may be due to its highly biased base composition and/or to regulatory signals or secondary structures that interfere with PCR amplification or DNA sequencing. It is in this region (*trnT-trnP-*CR-*trnF-rrnS*) that eight of these mitochondrial genomes are clearly incompletely sequenced, despite the statements to the contrary in the GenBank records. Figure 2 shows the sequence, approximately to scale, for this region in these eight mtDNAs compared to that otherwise typical of mammalian mtDNA. Each has a missing or incomplete *trnF*. Five have a missing *trnP* and, of these, three have an incomplete *trnT*. Five have no sequence in the region expected as the CR between these tRNAs and the other three have much shorter sequence than is typical. Others with unusually short CRs, but fully formed flanking tRNA genes could also be incomplete, especially considering these results, although it is not possible from these records to determine potential deletions.

In some cases, there is no annotation of a D-loop region (which is an optional miscellaneous feature), but sequence is present that is likely to serve that role. This is the case, for example, for seven species of bears in genera *Helarctos*, *Melursus*, *Tremarctos*, and *Ursus*, which have pairwise nucleotide sequence identities to the region annotated as D-loop of *Arctodus simus* of at least 57%. This is also the case for other carnivores, *Canis lupus lupus*, *Gulo gulo*, and *Phoca vitulina*.

**Fig. 3:**
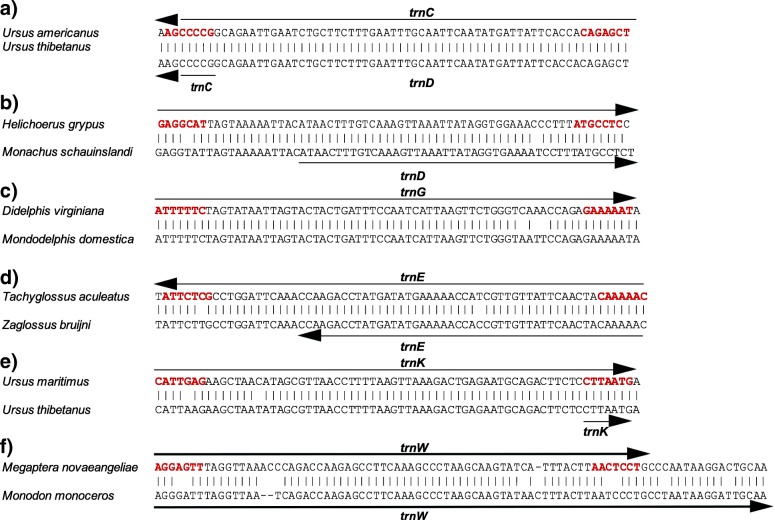
Errors of annotation on tRNA genes in mammalian mitochondrial genome. Alignments with closely related species and annotations as in the GenBank records to illustrate that the endpoints are incorrectly annotated for **a**) *trnC* in *Ursus thibetanus*, **b**) *trnD* in *Monachus schauinslandi,* **c**) *trnG* in *Monodelphis domestica,* **d**) *trnE* in *Zaglossus bruijni* (and by just one nucleotide in *Tachyglossus aculeatus*), **e**) *trnK* in *Ursus thibetan*, and *us* and f) *trnW* in *Monodon monoceros* Nucleotides shown in red boldface are those predicted to be paired in the amino-acyl acceptor arm of the mature tRNA

Further, there are four cases where a too short gene annotation (couldn’t be functional.) is easily seen to be in error, although the correct gene is designated: *trnC* in *Ursus thibetanus*; *trnD* in *Monachus schauinslandi*; *trnG* in *Monodelphis domestica*; *trnE* in *Zaglossus bruijni*, and *trnK* in *Ursus thibetanus* (Fig. 3). *trnW* in *Monodon monoceros* is annotated in error by being too long at its 3′ end, and there is no annotation of *trnG* in *Monodelphis domestica*, despite that tRNA gene being in the expected position with high sequence similarity to those of other species (Fig. 3). This suggests that the additional form of errors where the gene endpoints are not correctly designated could be common, but we have made no systematic effort here to identify them across these many mitochondrial genomes.

## Discussion

Our conclusion is that all of these 304 mitochondrial genomes (other than a few slight ambiguities due to incomplete sequencing; see above) have one of two arrangements. All of the monotremes and all of the eutherians share one arrangement. This is differentiated from the rearrangement of the “W-A-N-C-Y” region to “-A-CW-N-Y” shared by the 30 marsupials. All of the other 68 cases of genes reported to differ in arrangements among mammals (Additional file 1: Table S1 and Additional file 2: Table S2) are errors of annotation in the GenBank records. The “-A-CW-N-Y” ordination has been confirmed in marsupials by PCR and sanger sequencing, as in the case of mitogenome of *Didelphis virginiana* [22].

**Fig. 4:**
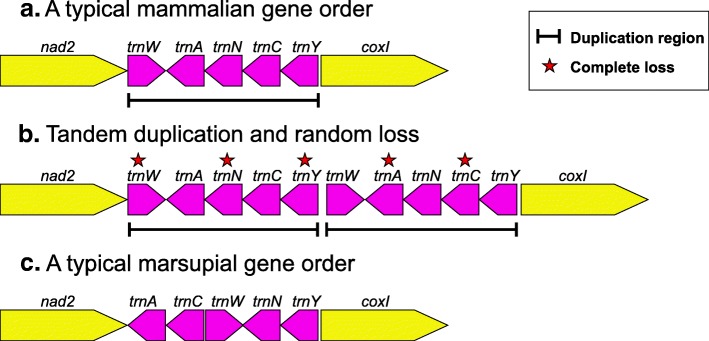
The hypothetical process of gene rearrangement in the model of tandem duplication/random loss for “WANCY” region in mammals. **a**. A typical mammalian “W-A-N-C-Y” gene order. **b**. Tandem duplication and random loss. The black lines indicate the duplicated region and the red stars show the genes lost during the evolution of marsupials. **c**. A typical marsupial gene order

Our results confirm that the mammalian mitochondrial genome is highly conserved in its gene order and that all of the rearrangements annotated in mammalian genomes other than the single one shared by all studied, extant marsupials, as observed in previous studies [3, 10, 22, 23], are annotation errors. The rearrangements within “W-A-N-C-Y” region between the order shared by monotremes and eutherians and the order shared by marsupials can be straightforwardly modeled using the “duplication/random loss” model (see [14]); as illustrated in Fig. 4.

Errors in genome annotation may be extensive, not only among the 9000 total mtDNAs already in GenBank, but in other genomes as well, especially as genome sequencing continues to accelerate and as erroneous annotations are sometimes used as the basis for further genome annotations, resulting in what has been called a “percolation of errors.” Despite all efforts of manual curation, it is still plagued by misassignments of reading directions, erroneous gene names, errors of gene endpoints, and missing as well as false positive annotations, in particular for the tRNA genes [24] and especially for annotating gene orientation. To prevent errors from spreading out of control, database curation by the scientific community will be essential [25]. However, much could be done simply by NCBI implementing scripts (see [16]) that could screen in automated fashion for the most obvious and common of errors, e.g., likely missing gene annotations or annotations of a gene on the wrong strand. Simply using BLAST in each orientation for each gene annotation would have easily flagged for closer inspection every gene arrangement error that was detected in this analysis.

## Conclusions

In summary, a significant number of rearrangements (68 of 187) in the mammalian mitochondrial genome were confirmed as errors of annotation, including false inversion in tRNAs and partial or complete deletions of tRNAs and D-loop region; confirming that the mammalian mitochondrial genome is preserved in its order. We further hope that curators at NCBI will correct these many errors in their database using this set of interpretations.

## Additional files

Additional file 1: **Table S1.** Analysis of gene arrangements of 304 mammalian mitochondrial genome. Gene arrangements of the mammalian species compared in this analysis as annotated at NCBI with Reference IDs shown for each. The numeral in parentheses indicates the number of species belonging to each group. Each gene is assigned a number 1–37 at the top of the Table which is then used to describe the annotated gene arrangement for each species. Each gene is transcribed left-to-right as shown except for those with a minus (−) symbol to indicate opposite orientation. The arrangement shared by all three monotreme and human mitochondrial genomes has long been inferred to be the ancestral condition for mammals. Highlighting indicates all deviations from that ancestral arrangement according to the NCBI sequence records, with green being confirmed by this work, red being errors of gene arrangement according to our analysis, brown being errors of gene boundary annotation according to our observation, orange being a failure to annotate a gene that we determined to be present, and gray being a gene missing wholly or in large part because the sequence is not present in the file. *trnY* for *Aotus nancymaae*, colored red here, is also in error for gene boundary errors. ^1^Extinct species. (XLSX 62 kb)

Additional file 2: **Table S2.** Lengths of each gene and of the total for each of the 304 mammalian mitochondrial genomes analyzed. “CR” is the control region that normally contains a D-loop structure, with the number in this column being all nucleotides annotated as the control region (or a synonym such as “A + T-rich region” or “D-loop region”) or, if lacking such annotation, the total number of unassigned nucleotides reported between annotations of flanking tRNA genes. The absence of any reported sequence between *trnP* and *trnF* is represented by zero (0). Highlighting follows the scheme in Table S2 with grey here also representing cases where there is no CR sequence in the GenBank record. *trnY* for *Aotus nancymaae*, colored red here, is also in error for gene boundary errors. The numeral inparentheses indicates the number of species whose mitogenomes were analyzed in each order. (XLSX 77 kb)

Additional file 3: **Figure S1.** Multiple alignment by MUSCLE of “ACWNY region” in twelve different marsupials in the order Diprotodontia. The orientation of genes according to the NCBI sequence annotation is represented by colored arrows. This highlights the evidence that *trnA* of *Dactylopsila trivirgata* is misannotated and, instead, is actually in the same orientation as the other marsupials. This analysis was performed on the Geneious software. (PDF 1735 kb)

## Abbreviations

“WANCY”: Region containing the tRNA genes *trnW, trnA, trnN, trnC, trnY*
*ATP*: Adenosine triphosphate
BLAST: Basic Local Alignment Search Tool
CR: Control region
D-loop: Displacement loop
mtDNA: Mitochondrial Deoxyribonucleic acid
MUSCLE: MUltiple Sequence Comparison by Log-Expectation
NCBI: National Center for Biotechnology Information
*OL*: Light-strand replication origin
