# Supplementary material for: Gene annotation errors are common in the mammalian mitochondrial genomes database
Source: BMC Genomics. 2019 Jan 22;20:73. doi: 10.1186/s12864-019-5447-1 (PMC6341679; doi:10.1186/s12864-019-5447-1)
Supplement: Supplementary file 3 — Figure S1. Multiple alignment by MUSCLE of “ACWNY region” in twelve different marsupials in the order Diprotodontia. The orientation of genes according to the NCBI sequence annotation is represented by colored arrows. This highlights the evidence that trnA of Dactylopsila trivirgata is misannotated and, instead, is actually in the same orientation as the other marsupials. This analysis was performed on the Geneious software. (PDF 1735 kb) [file 12864_2019_5447_MOESM3_ESM.pdf]

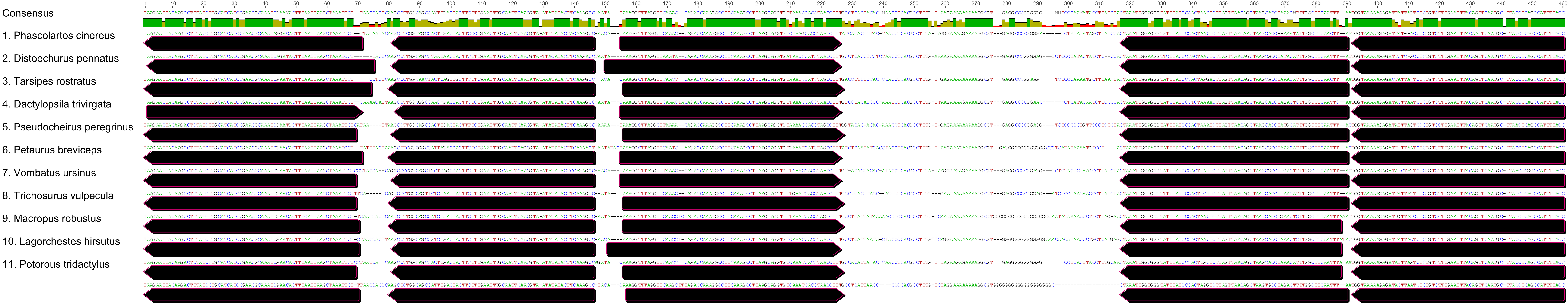
Consensus
1. Phascolartos cinereus
2. Distoechurus pennatus
3. Tarsipes rostratus
4. Dactylopsila trivirgata
5. Pseudocheirus peregrinus
6. Petaurus breviceps
7. Vombatus ursinus
8. Trichosurus vulpecula
9. Macropus robustus
10. Lagorchestes hirsutus
11. Potorous tridactylus
